# MAST: Phylogenetic Inference with Mixtures Across Sites and Trees

**DOI:** 10.1093/sysbio/syae008

**Published:** 2024-02-29

**Authors:** Thomas K F Wong, Caitlin Cherryh, Allen G Rodrigo, Matthew W Hahn, Bui Quang Minh, Robert Lanfear

**Affiliations:** School of Computing, Australian National University, Canberra, ACT 2601, Australia; Research School of Biology, Australian National University, Canberra, ACT 2601, Australia; School of Biological Sciences, University of Auckland, Auckland 1142, New Zealand; Department of Biology and Department of Computer Science, Indiana University, Bloomington, Indiana 47405, USA; School of Computing, Australian National University, Canberra, ACT 2601, Australia; Research School of Biology, Australian National University, Canberra, ACT 2601, Australia

**Keywords:** Incomplete lineage sorting, introgression, mixture model, multitree model, phylogenetics

## Abstract

Hundreds or thousands of loci are now routinely used in modern phylogenomic studies. Concatenation approaches to tree inference assume that there is a single topology for the entire dataset, but different loci may have different evolutionary histories due to incomplete lineage sorting (ILS), introgression, and/or horizontal gene transfer; even single loci may not be treelike due to recombination. To overcome this shortcoming, we introduce an implementation of a multi-tree mixture model that we call mixtures across sites and trees (MAST). This model extends a prior implementation by Boussau et al. (2009) by allowing users to estimate the weight of each of a set of pre-specified bifurcating trees in a single alignment. The MAST model allows each tree to have its own weight, topology, branch lengths, substitution model, nucleotide or amino acid frequencies, and model of rate heterogeneity across sites. We implemented the MAST model in a maximum-likelihood framework in the popular phylogenetic software, IQ-TREE. Simulations show that we can accurately recover the true model parameters, including branch lengths and tree weights for a given set of tree topologies, under a wide range of biologically realistic scenarios. We also show that we can use standard statistical inference approaches to reject a single-tree model when data are simulated under multiple trees (and vice versa). We applied the MAST model to multiple primate datasets and found that it can recover the signal of ILS in the Great Apes, as well as the asymmetry in minor trees caused by introgression among several macaque species. When applied to a dataset of 4 Platyrrhine species for which standard concatenated maximum likelihood (ML) and gene tree approaches disagree, we observe that MAST gives the highest weight (i.e., the largest proportion of sites) to the tree also supported by gene tree approaches. These results suggest that the MAST model is able to analyze a concatenated alignment using ML while avoiding some of the biases that come with assuming there is only a single tree. We discuss how the MAST model can be extended in the future.

Molecular phylogenetics aims to infer phylogenetic trees, often from aligned DNA or amino acid (AA) sequencing data. Many popular phylogenetic tools are designed to infer a single tree from a multiple sequence alignment, using one of a number of approaches such as ML (e.g., RAxML (Stamatakis 2014), IQ-TREE (Kalyaanamoorthy et al. 2017), and PhyML (Guindon et al. 2010)), Bayesian inference (e.g., MrBayes (Ronquist and Huelsenbeck 2003) and BEAST (Bouckaert et al. 2019)), maximum parsimony (e.g., MPBoot (Hoang et al. 2018), matOptimize (Ye et al. 2022), and TNT (Goloboff and Catalano 2016)), or distance methods (e.g., BioNJ (Gascuel 1997), FastME (Lefort et al. 2015), QuickTree (Howe et al. 2002), and RapidNJ (Simonsen and Pedersen 2011)). The assumption that the data can be represented as a single tree is appropriate when analyzing a single non-recombining locus. However, there are many situations where this “treelikeness” assumption is violated. For example, an alignment of a single locus may contain one or more recombination events in its history, such that different regions of the alignment follow different trees. More generally, it is well known that different genomic loci may have evolved under different trees due to biological processes including incomplete lineage sorting (ILS), hybridization/introgression, and horizontal gene transfer (Maddison 1997; Nichols 2001). Since modern phylogenomic datasets now routinely contain hundreds or thousands of loci, a great deal of work has focused on developing methods and software that relax the treelikeness assumption (Edwards 2009).

Most existing approaches that account for complex histories in large datasets focus on inferring either species trees or species networks, either from a single concatenated alignment or from many individual locus alignments or individual locus trees. Many of the most popular approaches for inferring species trees are based on the multi-species coalescent model (MSC) or are consistent with the MSC, and can infer a species tree while accounting for ILS among loci (e.g., SNAPP (Bryant et al. 2012), ASTRAL-III (Zhang et al. 2018b), MP-EST (Liu et al. 2010), SVD-Quartets (Chifman and Kubatko 2015), *BEAST (Heled and Drummond 2010), and *BEAST2 (Ogilvie et al. 2017)). More recent work has extended the MSC to account for a broader range of processes that can cause reticulations in the underlying species tree. These methods use models referred to as the multi-species network coalescent (or MSNC) and typically infer a species network that represents both the vertical inheritance and horizontal exchange of genetic material among evolving lineages (e.g., PhyloNet (Wen et al. 2018), PhyloNetworks (Solís-Lemus et al. 2017), SpeciesNetwork (Zhang et al. 2018a), and BPP (Flouri et al. 2018)). Other methods, like Relate (Speidel et al. 2019) and tsinfer (Kelleher et al. 2019), infer multiple tree topologies (as an approximation of an ancestral recombination graph) along genomes, although these methods are designed for within-species analyses.

In this study, we present a different solution to the problem of accounting for multiple histories in a single sequence alignment: the mixtures across sites and trees (MAST) model. The MAST model is an example of a *multitree* mixture model (Boussau et al. 2009; Allman et al. 2012) because it uses mixtures of bifurcating trees to represent the multiple histories present in a dataset. In phylogenetic mixture models, a number of sub-models (known as classes) are estimated from the data, and the likelihood of each site in the alignment is calculated as the weighted sum of the likelihood for that site under each sub-model (Fig. 1). Mixture models have been widely used in phylogenetic inference, including in rate heterogeneity across site models (Yang 1994), (Kalyaanamoorthy et al. 2017), profile mixture models (e.g., the CAT model (Lartillot and Philippe 2004)), mixtures of substitution rate matrices (e.g., the LG4M and LG4X models (Le et al. 2012)), and mixtures of branch lengths (e.g., the GHOST model (Crotty et al. 2020)).

**Figure 1:**
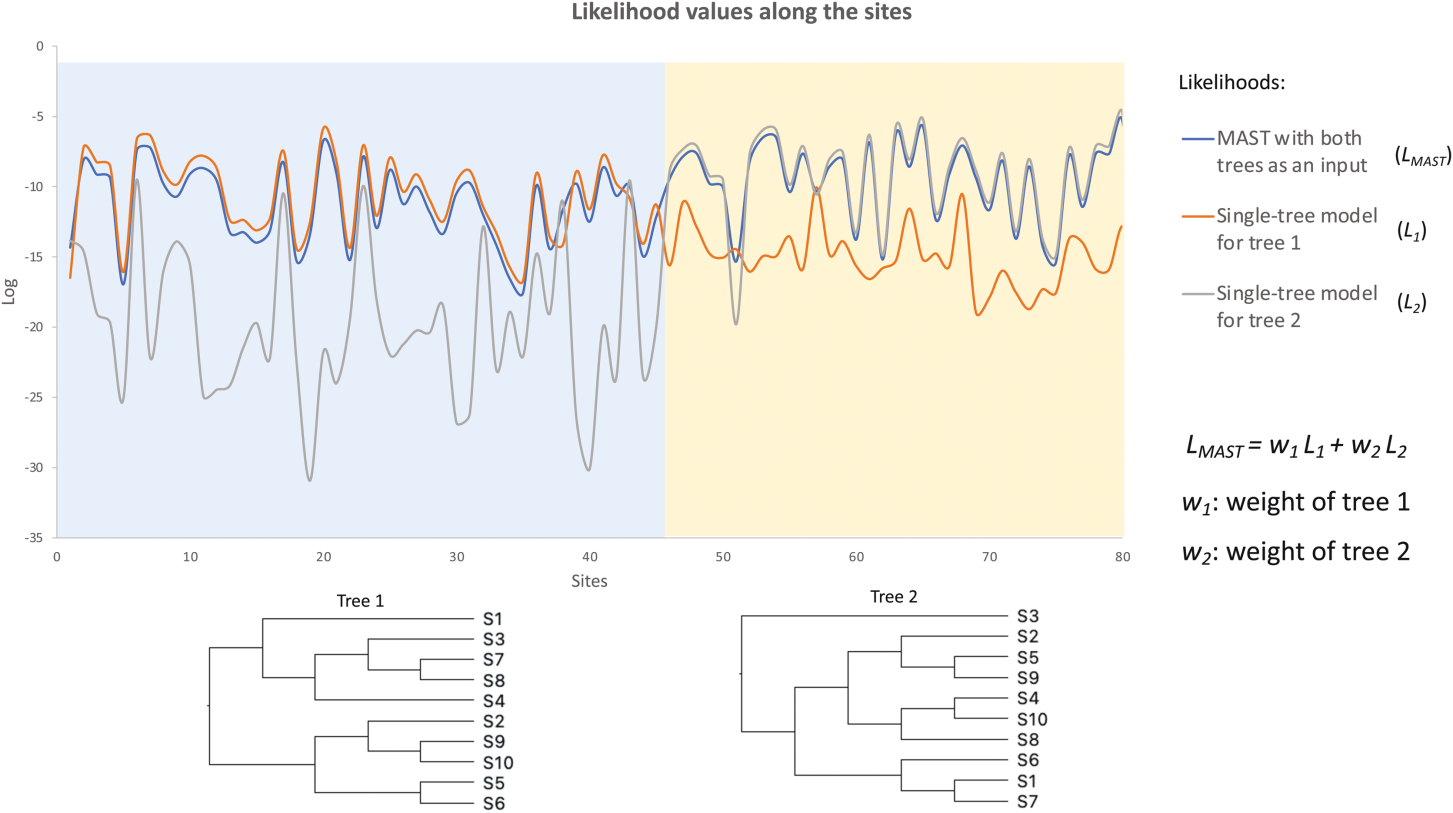
An example illustrating the MAST model. Two regions (of length 45 bp and 35 bp) were simulated under 2 different topologies, each with 10 taxa. The curves at the top show the site likelihoods (on a log scale) computed under tree 1 ($L_{1}$), tree 2 ($L_{2}$), and the MAST model ($L_{MAST}$). $L_{MAST}$ is calculated as the weighted sum of $L_{1}$ and $L_{2}$, where the weight parameters $w_{1}$ and $w_{2}$ will be estimated by the MAST model. This toy example shows that the $L_{MAST}$ curve matches the $L_{1}$ curve for region 1 and the $L_{2}$ curve for region 2 with high site likelihoods, demonstrating the ability of the MAST model to predict the true underlying evolution of this data. Note that due to the log scale of the y-axis, the log value of *L*_*MAST*_ is much closer to the log value of the higher likelihood value between *L*_*1*_ and *L*_*2*_.

Multitree mixture models are best seen as a generalization of a standard concatenated phylogenetic analysis. In a standard concatenated phylogenetic analysis, we assume that the history of the entire alignment is represented by a single bifurcating phylogenetic tree (i.e., we make the treelikeness assumption). Multitree mixture models relax this assumption and represent the history of the alignment with a mixture of any number of tree topologies. The MAST model is similar to a previous implementation of a multitree mixture model, PhyML-multi (Boussau et al. 2009). Crucially, though, it estimates the weights of the input trees from the data, while PhyML-multi assumes that all trees have equal weights. In addition, MAST implements the full range of models available in IQ-TREE2 and gives users flexible options for how to associate different aspects of the evolutionary models with the different trees. Given an alignment and a collection of tree topologies that contain the same tip labels as that alignment, the MAST model estimates the likelihood of each site under each tree, the maximum-likelihood weights of each of the input trees, the branch lengths of the trees, and the other free parameters of the substitution model. In this way, it has many of the advantages of concatenation approaches but can accommodate underlying discordance in the alignment (Bryant and Hahn 2020).

The multitree mixture model implemented in MAST differs from species tree and species network models in a number of ways. As opposed to many MSC and MSNC approaches, the MAST model does not explicitly model biological processes such as ILS, introgression, or horizontal gene transfer. Instead, the MAST model is process-agnostic and simply seeks to calculate the relative weights of tree topologies from the input data. This is a limitation in the sense that the output of the MAST model does not contain direct estimates of many evolutionary parameters of interest, such as the number of hybridization events, their location on the species tree, or ancestral population sizes. Similarly, just as with standard single-tree concatenation approaches, the MAST model cannot represent distributions of branch lengths on a single tree topology, as expected under the coalescent. On the other hand, that MAST is process-agnostic may be seen as a strength because the MAST model can represent a wide range biological processes (e.g., differences in tree topologies caused by the coalescent or by introgression) or technical errors (such as the accidental inclusion of paralogs) that can cause the treelikeness assumption to be violated. Moreover, the MAST model differs from previous approaches because it calculates the likelihood of every site under every tree in the mixture while estimating the weights of the input trees from the data. Although these weights are not equivalent to gene-tree frequencies, they may in practice be quite similar in value. Similarly to some implicit network models, MAST assumes that sites are independent of one another. In other words, the order of the sites in the alignment will not affect the parameter estimates from the MAST model. This means that MAST is agnostic with respect to the underlying rate at which tree topologies change along an alignment. As with other aspects of MAST, this makes it a relatively general model, but at the cost of ignoring the potentially useful information contained in many alignments that arises from the fact that neighboring sites often share the same tree topology. Our simulations demonstrate that the MAST model accurately recovers tree weights even when neighboring sites are highly correlated in their association with tree topologies (see below).

In this paper, we first describe the mathematical basis of the MAST model and its implementation in IQ-TREE. This implementation allows us to estimate tree weights, model parameters, and branch lengths for a given set of input tree topologies. We then perform extensive simulations to evaluate the accuracy and the limitations of the MAST model. Finally, we demonstrate the use of the MAST model on 4 empirical datasets of primates to show that it recapitulates results from well-studied clades. We also highlight the advantages of MAST over standard phylogenetic analysis methods when applied to these datasets.

## Material and Methods

### The MAST Model

In a standard concatenated maximum likelihood (ML) analysis (such as that performed by IQ-TREE (Nguyen et al. 2015) or RAxML (Stamatakis 2014)), it is assumed that every site in the concatenated alignment comes from a single phylogenetic tree, which consists of a topology and branch lengths. In this framework, ML approaches seek to find the model of sequence evolution, tree topology, and branch lengths that maximize the likelihood of the observed alignment. The MAST model generalizes this framework by assuming that each site in the alignment comes from a mixture of m trees. Each tree has its own weight, topology and branch lengths, and the trees may have independent or shared substitution models (e.g., the general time reversible (GTR) model (Tavaré 1986)), a set of nucleotide or amino-acid frequencies, and a rate heterogeneity across sites (RHAS) model (e.g., the +G or +I+G models). In what follows, we first describe the case in which each tree has an independent substitution model, set of nucleotide or AA frequencies, and RHAS model.

### Model Description

The MAST model consists of m classes where each class *j* comprises a bifurcating tree topology $T_{j}$. For the *j*th class, $\lambda_{j}$ is defined as the set of branch lengths on $T_{j}$, $R_{j}\;$ as the relative substitution rate parameters, $F_{j}$ as the set of nucleotide or amino-acid frequencies, $H_{j}\;$ as the rate heterogeneity model, and $w_{j}$ as the class weight ($w_{j}>0$, $\overset{m}{\underset{j=1}{\sum }}w_{j}=1$). Given a multiple sequence alignment, A, we define $L_{ij}$ as the likelihood of the data observed at *i*th site in A under the *j*th class of the MAST model. $L_{ij}$ can be computed using Felsenstein’s pruning algorithm (Felsenstein 1981). The likelihood of the *i*th site, $L_{i}$, is the weighted sum of the $L_{ij}$ over the m classes:


$$\begin{array}{l} L_{i}=\sum_{j=1}^{m}w_{j}L_{ij}(T_{j},\lambda_{j},R_{j},H_{j},F_{j}). \end{array}$$ (1)

The full log-likelihood l over all N alignment sites, which are assumed to be independent and identically distributed (iid), is:


$$\begin{array}{l} l=\sum_{i=1}^{N}log(L_{i})=\sum_{i=1}^{N}log\left(\sum_{j=1}^{m}w_{j}L_{ij}\left(T_{j},\lambda_{j},R_{j},H_{j},F_{j}\right)\right). \end{array}$$

This formula is very similar to the formulation of the GHOST model (Crotty et al. 2020) and the PhyML-multi (Boussau et al. 2009). The GHOST model allows for mixtures of branch lengths on a single topology and differs only insofar as the final sum here is across the m tree topologies and their associated branch lengths, versus the m sets of branch lengths on a single topology in the GHOST model. The PhyML-multi model assumes the same probability across all the trees, whereas the MAST model generalizes this and allows different probabilities by introducing the tree weight ($w_{j}$) parameters.

In the implementation of the MAST model we describe here, we assume that we know the topologies of all of the m trees ahead of time, for example, the set of gene tree topologies observed among the genomes, or the set of possible trees that should appear under the MSC model. We then estimate the relative weights (i.e., proportions) of each topology, optimize the branch lengths of each topology, the parameters of the evolutionary model, and the nucleotide or amino-acid frequencies for each tree. We consider extensions of the model when the tree topologies are not given in the “Discussion” section.

### Linked and Unlinked MAST Submodels

**Figure 2:**
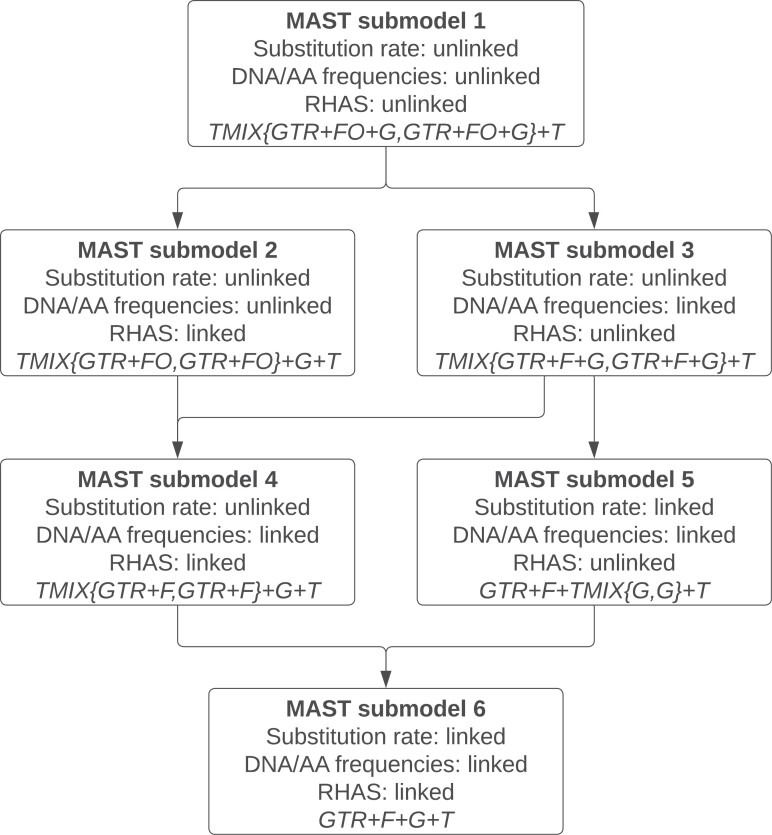
A hierarchy of 6 MAST submodels currently implemented in IQ-TREE. The term “unlinked” means the parameters can differ across mixture classes, while “linked” means the parameters are restricted to be equal across all classes. The last line in each box shows the name of the model that can be used directly as input in IQ-TREE via -m option, assuming two classes with a GTR substitution model and Gamma RHAS model for each class. The arrows indicate the nestedness between the submodels; for example, submodel 4 is nested within both submodels 2 and 3, while submodel 6 is nested within both submodels 4 and 5. Note that two submodels are missing (i.e., substitution rate: linked; DNA/AA frequencies: unlinked; RHAS: linked/unlinked) due to a non-trivial implementation.

In standard phylogenetic analyses we estimate a single tree with an associated set of branch lengths, along with the parameters of the substitution model, the base or AA frequencies, and the RHAS model. In the most general MAST model introduced above (submodel 1 in Fig. 2), the tree, the branch lengths of that tree, the substitution model, the base or AA frequencies, and the RHAS model can all vary in each class, and the weight of that class pertains to the full set of free parameters associated with that class. We say that all parameters are unlinked across classes in this model. We also allow for 5 more-restrictive models in which the parameters of the substitution models, the vectors of base or AA frequencies, or the RHAS model can be linked across all m classes of trees. The most restricted model (submodel 6 in Fig. 2) links the parameters of all 3 of these components of the model across all m classes of trees. In this model, the estimated weights, therefore, pertain *only* to the trees and their branch lengths in each of the m classes, because these are the only parameters allowed to differ among classes. This framework allows for the comparison of models with likelihood ratio tests or other information criteria (Burnham and Anderson 2002).

### Model Parameter Estimation for Fixed Topologies

**Figure 3:**
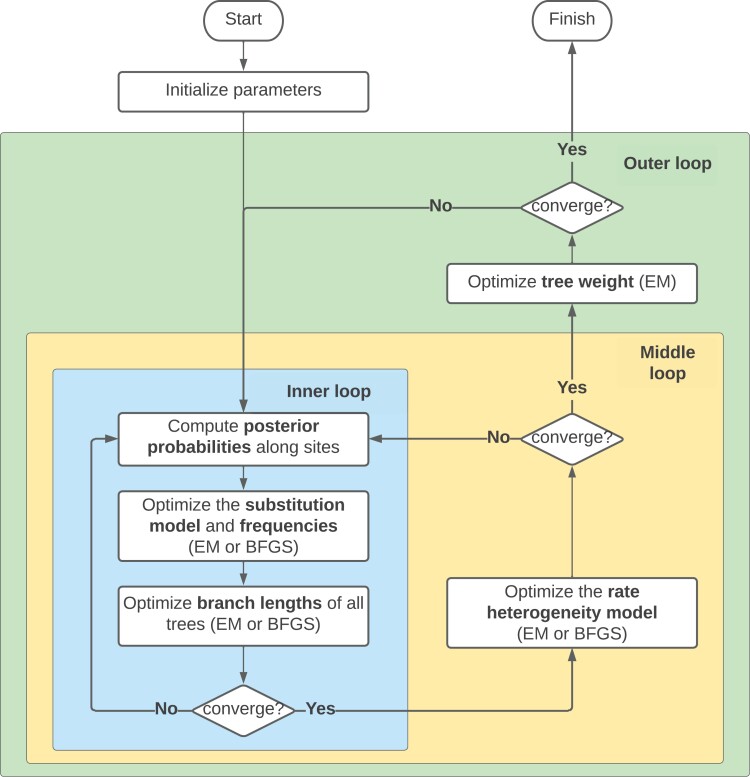
Optimization flow chart for the MAST model in IQ-TREE. The optimization workflow includes an outer loop, a middle loop, and an inner loop of iterations. The inner loop optimizes the substitution model, nucleotide frequencies, and branch length of the trees; the middle loop optimizes the rate heterogeneity model; the outer loop optimizes the tree weights. The EM algorithm is used to optimize the individual unlinked parameters of each tree and the BFGS algorithm is used to optimize the linked parameters. The iterations continue until the likelihood value converges.

Given a set of fixed topologies, $T_{1},\ldots ,T_{m}$, the challenge is to optimize all of the parameters without getting stuck in local optima. We employ both the expectation-maximization (EM) algorithm (Dempster et al. 1977) and the Broyden–Fletcher–Goldfarb–Shanno (BFGS) algorithm (Fletcher 2013) to estimate the MAST model parameters. Taking advantage of the existing parameter optimization algorithms implemented in IQ-TREE, our workflow (Fig. 3) operates as follows. To begin, for class j, the substitution model $R_{j}$ and the nucleotide or amino-acid frequencies $F_{j}$ are initialized as a Jukes–Cantor model (i.e., $\hat{R_{j}}=1$ and uniform frequencies $F_{j}$), and the branch lengths $\lambda_{j}$ are initialized as the maximum parsimony (Fitch 1971) branch lengths of the tree $T_{j}$. To obtain some sensible initial values of the tree weights, we first compute the parsimony scores for each tree topology along all the sites. For each of the sites with different parsimony scores between the tree topologies, we then check which tree topology has the minimum parsimony score and assign the site to that tree. The tree weights are then initialized according to the proportion of these sites assigned to each of the trees. If all sites have the same parsimony scores across all the trees, then the tree weights are initialized to be equal.

Having established the starting values for all the parameters in the model, we then optimize them. The optimization of each class of model parameters is done sequentially. Figure 3 summarizes the workflow of the optimization. Our optimization workflow includes an outer loop, a middle loop, and an inner loop of iterations. The inner loop optimizes the substitution model, nucleotide frequencies, and branch length of the trees; the middle loop optimizes the rate heterogeneity model; the outer loop optimizes the tree weights. This optimization continues to iterate until the resulting log-likelihood value converges, where convergence is defined as the increment of the log-likelihood value in the current iteration falling below some threshold ϵ (which we set to 0.0001). To optimize the unlinked parameters of each tree in the mixture model, we use an EM algorithm similar to that used in the GHOST model (Crotty et al. 2020).

In detail, our calculations are as follows. Define $p_{i,j}$ as the posterior probability of site $D_{i}$ evolving under a tree $T_{j}$. The value of $p_{i,j}$ is computed by the following equation:


$$\begin{array}{l} p_{i,j}=\frac{w_{j}L_{ij}(T_{j},\lambda_{j},R_{j},H_{j},F_{j})}{\overset{m}{\underset{j=1}{\sum }}w_{j}L_{ij}(T_{j},\lambda_{j},R_{j},H_{j},F_{j})}. \end{array}$$

The expectation of the log-likelihood value ($l_{j}$) of tree *j* over all the sites:


$$\begin{array}{l} E\left[l_{j}\right]=\overset{N}{\underset{i=1}{\sum }}p_{i,j}log(L_{ij}(T_{j},\lambda_{j},R_{j},H_{j},F_{j})). \end{array}$$

In every iteration, by fixing the posterior probabilities $p_{i,j}$, we optimize the tree weights, the branch lengths, the unlinked substitution rate models, and the unlinked rate heterogeneity models of all trees one-by-one to maximize the expected likelihood value. The tree weights are then updated by averaging the probabilities over all the N sites. That is, the new weight of class j is the mean posterior probability of each site belonging to class j:


$$\begin{array}{l} w_{j}=\frac{1}{N}\overset{N}{\underset{i=1}{\sum }}p_{ij}. \end{array}$$ (2)

For the linked models (submodels 2–6 in Fig. 2), the EM algorithm cannot be applied to the optimization of the linked parameters shared between the classes. Thus, we optimize the parameters of the linked substitution rate model *R*, the linked nucleotide or AA frequencies *F*, and the linked rate heterogeneity model *H* using the BFGS algorithm in IQ-TREE.

## Simulations

Having implemented the MAST model in IQ-TREE, we next used simulated data to test the performance of the MAST model under a wide range of scenarios. The first and second simulation experiments test the accuracy of the unlinked and linked MAST models when the true model is specified. We also compared the performance between the MAST model and the PhyML-multi model when all trees have unlinked parameters. The third simulation experiment simulates data with varying levels of introgression to compare the performance of standard (i.e., single-tree) concatenation methods to the performance of the MAST model. The fourth and fifth simulation experiments examine the performance of the MAST model when an incorrect model is specified, by applying an unlinked and linked MAST model with different numbers of trees to an alignment simulated under a single tree. The sixth simulation experiment evaluates the performance of the MAST model when all possible tree topologies are provided for the input alignment.

### Simulations 1 and 2: Parameter Estimation Under the True Model for Unlinked and Linked MAST Model (Submodel 1 and Submodel 6)

These simulations are designed to ask whether our implementation of the MAST model in IQ-TREE is capable of estimating accurate tree weights, branch lengths, and other model parameters when the model used for inference matches the model used for simulation. We simulated alignments under the completely unlinked MAST model (submodel 1 in Fig. 2; simulation 1) and the completely linked MAST model (submodel 6 in Fig. 2; simulation 2), and provided IQ-TREE with the set of true tree topologies from the mixture, as well as the true model of molecular evolution (e.g., GTR + G), and the correct MAST model (i.e., submodel 1 or 6). We then measured the accuracy of our implementation by recording the estimated tree weights, branch lengths, substitution model parameters, and nucleotide frequencies, and comparing them to the values used to simulate the data.

We simulated alignments from mixtures of *m* of trees with different numbers (*t*) of taxa, where m∈{1,2,3,5,10} and t∈{6,7,10,20}. We performed 100 replicate simulations for every combination of *m* and *t*, for a total of 2000 simulated datasets per experiment.

Different GTR model *R*, gamma rate *H*, and set of nucleotide frequencies *F* were simulated over the trees in the first simulation experiment, while the same *R*, *H*, and *F* were shared among the trees in the second simulation experiment. The alignments were then simulated according to the tree, the GTR model, and the gamma rate using AliSim (Ly-Trong et al. 2022).

Each simulated dataset contained 100K bases, regardless of the number of trees *m*, with different proportions of the lengths of each of the *m* alignments. For clarity, details of how the model parameters were chosen are described in supplementary material.

To assess the accuracy of the parameter estimates, we calculated the root-mean-squared error (RMSE) of each estimated parameter when compared to its value in the simulation. For each dataset, we compared the statistical fit of the MAST model to that of a standard single-tree model by comparing the BIC value (BIC) of the MAST model to the BIC value (BIC0) of a standard single-tree model.

We did additional simulations to compare the performance of MAST to that of PhyML-multi, and to assess the accuracy of MAST on smaller alignments. To do this we repeated Simulation 1 with alignments of 5K, 10K, and 50K bases, and analyzed them with both PhyML-multi and MAST, both with unlinked parameters (i.e., each tree has its own GTR and +G models), as above. We evaluated both the multitree mixture and the HMM models of PhyML-multi. To assess the accuracy of the PhyML-multi HMM models (which do not compute tree weights), we calculated the RMSE between the proportion of sites assigned to each topology and the actual proportion of sites simulated from each topology.

### Simulation 3: Introgression

To examine the performance of the MAST model in a biologically motivated setting, we simulated alignments on 4-taxon trees with different levels of introgression and then used both a single-tree model and the linked MAST model (i.e., submodel 6) to analyze them. Each dataset was simulated from a rooted 4-taxon tree shown in Supplementary Fig. 8A. Using this tree, we simulated 1500 gene trees with introgression rate *r* from lineage 2 to lineage 4 (Supplementary Fig. 8A) using the program *ms* (Hudson 2002), where r∈{0.0, 0.1, 0.2,..., 0.9, 1.0} . When the introgression rate is zero, the largest fraction of the gene trees will match the species tree *T*_*E1*_ and the frequency of the two minor trees, *T*_*E2*_ and *T*_*E3*_, are expected to be equal. As the introgression rate increases, the frequency of the tree matching the introgression history, *T*_*E2*_, will increase, and the frequency of the other two trees will decrease. The MAST model should reflect these patterns in the tree weights calculated from a concatenated alignment of all 1500 genes, without the need to know the boundaries between the individual loci. The benefit of this approach when applied to an empirical dataset is that it overcomes concerns about “concatalesence,” in which unaccounted-for recombination within loci can bias estimates of gene tree frequency calculated by building trees for each locus (Gatesy and Springer 2014). Since *ms* uses a coalescent model, we rescaled the branch lengths from coalescent units to units appropriate for simulating alignments (i.e., substitutions per site) by multiplying all branch lengths by 0.002, selected to result in branch lengths similar to those recovered from our analyses of empirical dataset 4 (see below). For each simulated gene tree, we used AliSim (Ly-Trong et al. 2022) to simulate a 1000 bp alignment using the GTR + G model with parameters equal to those reported by IQ-Tree for our analysis of empirical dataset 4 (see below). Concatenating all the single-locus alignments resulted in an alignment of 1,500,000 bp. We performed 100 replicate simulations at every *r*, for a total of 1100 simulated datasets. We then applied the linked MAST model (submodel 6 in Fig. 2) to these data, with the input trees comprised of all 3 possible unrooted trees of the 4 taxa in Supplementary Fig. 8B.

### Simulations 4 and 5: Parameter Estimation Under Misspecified Models (Submodel 1 and Submodel 6)

We next sought to examine the performance of the MAST model when the underlying data were simulated under a single tree T, but the data were analyzed under a MAST model with m>1_,_ that is, a misspecified model with more than one tree. To do this, we simulated data under a single tree topology, and then applied MAST submodel 1 (simulation 4) and MAST submodel 6 (simulation 5) where the m trees included the true tree T and also m−1 additional tree topologies that differed from T. This simulation is designed to examine the case where a researcher includes the primary tree in a MAST model (e.g., a tree derived from a single-tree concatenated ML analysis, or an MSC analysis) but additionally includes some hypothesized trees in the model that have no support in the underlying data.

In simulation 4, we simulated alignments of 5K, 10K, and 50K bases, on a single tree with different numbers (*t*) of taxa, where t∈{6,7,10,20}. We performed 100 replicate simulations at every length and every *t*, resulting 300 simulated datasets for each *t*. To simulate each of the additional m−1 tree topologies in each MAST model, we sequentially performed k random subtree pruning and regrafting (SPR) moves on the true tree T. The MAST submodel 1 was then applied by inputting the actual tree topology as well as the other m−1 different tree topologies that all are k-SPR moves from that tree, where m∈{2,3,5,10}  and k∈{1,2,3}. Note that there are at most two SPR moves between any two 6-tip trees. Analyzing each of the 300 simulated datasets for 6-tip trees under 8 combinations of *m* and *k*, and each of a total of 900 simulated datasets for 7/10/20-tip trees under 12 combinations of *m* and *k*, gives a total of 13,200 analyses.

To understand the performance of the MAST model for submodel 6 under similar simulation conditions (simulation 5), we simulated data with the same settings as above, except that we used alignments of 100K bases.

To evaluate the performance, among the 100 replicates, we recorded how many times the true topology had the maximum tree weight. We also compared the BIC value (BIC) reported by the MAST model with the BIC value (BIC0) under the true model, that is, when the dataset was analyzed under the single true tree T.

### Simulation 6: Parameter Estimation When all Tree Topologies are Provided

We next evaluated the performance of the MAST model when all possible tree topologies are provided by the user, but the data were simulated on a smaller number of trees. To do this, we simulated data sets under two random equally weighted 5-tip trees with MAST submodel 6. We then applied the same MAST submodel, but with all 15 potential topologies of 5 taxa, to the data sets. This simulation is designed to examine the case where a researcher includes all possible hypothesized trees in the model, but that many of them in fact have no support in the underlying data. Each simulated dataset comprised 100K base pairs, and 100 replicate simulations were performed for each simulation setting. In order to further understand how BIC value of a MAST model depends on the input trees, after the above simulation we first fit a MAST submodel 6 with the two true trees, and we then fit a series of MAST submodel 6 with additional trees added sequentially based on the descending order of tree weights from the previous analysis involving all 15 trees. We recorded the BIC value of every model.

## Applications to empirical data

In addition to testing the MAST model on simulated data, we also applied it to four empirical datasets (Table 1). All of these datasets are subsets of a single dataset comprising 1730 single-gene alignments from 26 primates (Vanderpool et al. 2020). The first two empirical datasets we used are simple 4-taxon datasets, in which it is trivial to supply the MAST model with all 3 possible unrooted trees, and for which the expected tree weights have been estimated in previous research. In the other two empirical experiments, a standard single-tree model was first used to infer a topology for every gene in the dataset. Then, the set (or subset) of most commonly inferred gene trees was used as the set of input topologies for the MAST model when analyzing a concatenated alignment of all the single-gene alignments. In order to find out whether the MAST model has a better fit to the data compared with the standard single-tree model, we analyzed multiple different submodels of MAST (Fig. 2). We compared the lowest BIC values from these models to the BIC value calculated using the standard single-tree model on the same alignments.

**Table 1:** The 4 empirical datasets analyzed here

Empirical datasets	Species	# of genes	Total length
A	*Homo sapiens, Pan troglodytes, Gorilla gorilla, Pongo abelii*	1595	1,618,506
B	*Macaca fascicularis, Macaca mulatta, Macaca nemestrina, Colobus angolensis palliatus*	1599	1,629,163
C	*Homo sapiens, Pan troglodytes, Gorilla gorilla,* *Macaca fascicularis, Macaca mulatta, Macaca nemestrina*	1556	1,576,852
D	*Callithrix jacchus, Aotus nancymaae, Saimiri boliviensis, Cebus capucinus imitator, Macaca mulatta*	1557	1,610,755

The first dataset (“A”) includes the well-studied 4-taxon grouping of human, chimpanzee, gorilla, and orangutan. Previous studies have shown that all 3 possible unrooted gene trees of 4 taxa (Fig. 6; orangutan is considered an outgroup to the other three species) are recovered from these data. These studies have shown that the accepted species tree, uniting humans and chimps, is the most frequent gene tree, with the two minor trees occurring in very similar frequencies, consistent with the action of only ILS during the divergence of these species (Ebersberger et al. 2007); however, different studies have reported different frequencies for the 3 possible gene trees. For example, an early study that analyzed 11,945 gene trees (Ebersberger et al. 2007) and a more recent study that analyzed 1730 gene trees (Vanderpool et al. 2020) found that 77% and 62% of gene trees respectively grouped humans and chimps, 12% and 20%, respectively, grouped chimps and gorillas, and 11% and 18%, respectively, grouped humans and gorillas. The discrepancies in these numbers reflect both the different data types and data quality available to each study, as well as differences in the methods used to reconstruct gene trees. However, both studies made the single-tree assumption for each individual gene locus; recombination within each locus violates this assumption. The MAST model avoids this assumption by using mixtures of trees. Although the tree weights reported by MAST pertain to the equations given above and are not designed to replace estimates of gene tree frequencies, in practice we expect both values to be similar on large empirical datasets, because both values will usually be heavily influenced by the proportion of sites in the genome that are associated with each of the trees of interest. Since the MAST model will be unaffected by concatalescence, we expect that estimates of tree weights from the MAST model to be more accurate than estimates of gene tree frequencies from previous studies where concatalescence has affected gene-tree frequency estimates. Regardless, we still expect the MAST model to report the highest weight for the tree grouping humans and chimps, and lower but approximately equal weights for the two minor trees.

The second empirical dataset (“B”) includes 3 species from the genus *Macaca* (*M. fascicularis, M. mulatta, M. nemestrina*) and the mandrill (*Colobus angolensis palliatus*), a clade in which a previous analysis found substantial evidence for introgression between *M. nemestrina* and *M. fascicularis (**Vanderpool et al. 2020*). Thus, for this dataset, we expect the MAST model to recover the highest weight for the accepted species tree uniting *M. fascicularis* and *M. mulatta* ($T_{B3}$ in Fig. 7), the second highest weight for the minor tree affected most by introgression (uniting *M. nemestrina* and *M. fascicularis*), and the lowest weight for the minor tree uniting *M. mulatta* and *M. nemestrina*.

The third empirical dataset (“C”) contains the 6 species (human, chimp, gorilla, and the 3 *Macaca* species) that represent the ingroups from the first two datasets. Since we have a *priori* information that suggests that all 3 possible rooted trees are possible for each of these ingroups, we applied a MAST model with 9 trees (Supplementary Fig. 9), where all 3 resolutions of each ingroup clade are paired with all 3 resolutions of the other ingroup clade. In principle, one should be able to draw similar conclusions from these 6-taxon datasets as one could from the 2 independent analyses of the 4-taxon datasets by summing the relevant tree weights (see below).

The fourth empirical dataset (“D”) focuses on the relationships among 4 Platyrrhine (“New World Monkey”) species: *Callithrix jacchus*, *Aotus nancymaae*, *Saimiri boliviensis*, and *Cebus capucinus imitator*, including *Maccaca mulatta* as an outgroup. There is disagreement about the species tree among the 4 focal taxa. Gene-tree-based analyses (Vanderpool et al. 2020) support a caterpillar tree in which *Aotus* is the sister group to a clade uniting *Saimiri* and *Cebus* ($T_{D1}$ in Supplementary Fig. 10). However, concatenated ML analysis fails to recover this tree, instead returning a symmetrical tree likely caused by a known inconsistency in ML methods when the underlying gene trees are highly discordant (Kubatko and Degnan 2007; Roch and Steel 2015; Mendes and Hahn 2018). The MAST model should in principle avoid statistical inconsistencies associated with the single-tree assumption because it explicitly accounts for the existence of multiple histories in an alignment. Thus, we sought to test the performance of the MAST model in this well-studied empirical test case. To do this, we applied a MAST model that included the 3 ingroup topologies that were most commonly found from the gene trees in a previous study (Supplementary Fig. 10; Vanderpool et al. 2020).

We analyzed each empirical dataset using the same approach. First, we filtered the original 1730 locus dataset to retain only those loci that were present in all of the selected species, which resulted in each dataset containing approximately 1600 loci and around 1.6 million base pairs (Table 1). We analyzed each dataset using standard single-tree concatenated ML analyses (using default settings in IQ-TREE2), as well as the 6 multitree mixture models described by the 6 submodels of the MAST model in Fig. 2, using the trees topologies described above as the input topologies for the MAST model. Finally, to facilitate comparisons with other quantities of interest, we calculated the following quantities for each of the input topologies: (i) the number of single-locus trees that match each topology, where each single locus tree was estimated with default parameters in IQ-TREE2; and (ii) the total number of base pairs assigned to each topology (summing across single-locus trees), (iii) the total number of variable sites assigned to each topology (summing across single-locus trees), and (iv) the total number of parsimony informative sites assigned to each topology (summing across single-locus trees).

## Results

### Simulations 1–3: The MAST Model Performs Well When the Model is Correctly Specified, With or Without Introgression

Our extensive simulations demonstrate that the unlinked (Supplementary Figs. 1 and 2) and linked (Supplementary Fig. 3) MAST models perform well when the model used for analysis matches that used to simulate the data set for the data sets with lengths 5K, 10K, 50K (for the unlinked MAST model), and length 100K (for both the unlinked and the linked MAST models). The error associated with all unlinked and linked models increases as the number of trees in the mixture increases, as the number of tips in the tree decreases, and as the sequence length decreases. This is expected because in our simulations we held the distribution of branch lengths constant. Thus, the amount of information available to estimate each parameter decreases (and thus the expected error increases) as the number of trees increases, as the number of tips in each tree decreases, and as the sequence length decreases. The key parameters of interest for the MAST models are the tree weights (top panel, Supplementary Figs. 1 and 3; Supplementary Fig. 2A–C). In the best-case scenario (comprised of 2 trees, each of which contains 20 taxa, and an alignment of 100K bases), the RMSE of the tree weights was very low, at around 0.001 for both the unlinked and linked models, while in the worst-case scenario (comprised of 10 trees, each of which contains 6 taxa, and alignments of 5K bases (for unlinked model) and 100K bases (for linked model) sites) the error was much higher, at around 0.05 for both the unlinked and linked models, although this is still acceptably low in absolute terms.

**Figure 4:**
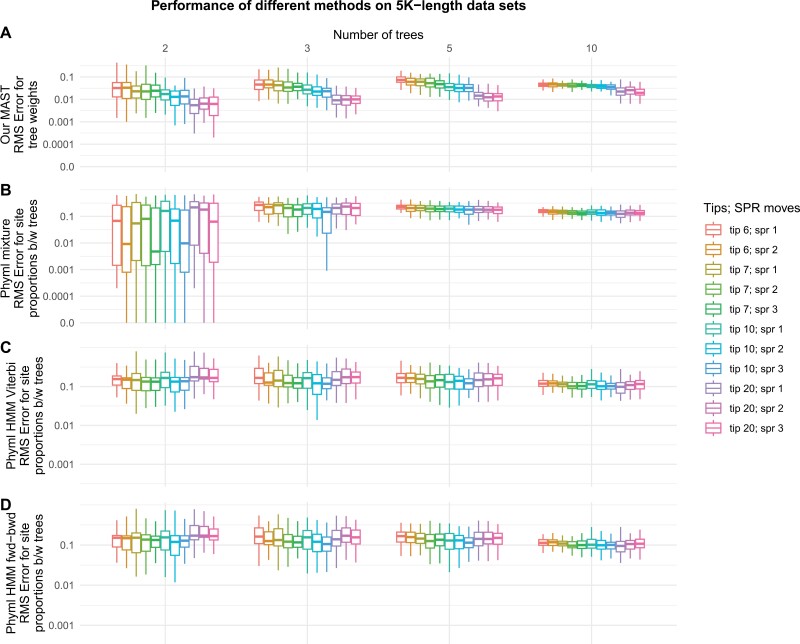
This figure illustrates the accuracy of tree weight estimates for the MAST model and the proportion of sites between the trees for the PhyML-multi software when the true topologies are provided, and the software was applied to 5K-length data sets simulated under the MAST model with unlinked parameters. Each tree has its own set of branch lengths, substitution matrices, nucleotide frequencies, and gamma parameters. The data sets were simulated with varying numbers of topologies (2, 3, 5, and 10) and numbers of sequences (6, 7, 10, and 20) in the alignments. Among the input trees, the first tree differed from the other trees by 1, 2, or 3 SPR moves. The root-mean-squared error (RMSE) distributions for these estimations are shown for (a) our MAST model, (b) PhyML-multi’s mixture model, (c) PhyML-multi’s HMM model with the Viterbi algorithm, and (d) PhyML-multi’s HMM model with the forward–backward algorithm. Note that PhyML-multi encountered errors when processing the 10K and 50K-length simulated data sets. On average, the RMSE reported by PhyML-multi, whether through the mixture or HMM model, exceed 0.1. In contrast, the RMSE for our MAST model remains below 0.1.

The simulation results (Fig. 4) comparing the performance between the MAST model and the PhyML-multi model illustrate that the MAST model performs better than the PhyML-multi model when the unlinked model used for analysis matches that used to simulate the data sets. On average, PhyML-multi reports RMSE exceeding 0.1, regardless of whether it uses the mixture model, HMM with the Viterbi algorithm, or HMM with the Forward–backward algorithm. In contrast, on average, our MAST model consistently reports RMSE well below 0.1. We were unable to compute model parameters with PhyML-multi on alignments longer than 5K bases because it reported undefined negative values (i.e., -nan) for the log-likelihoods of the models on alignments of 10K bases or longer.

The MAST model fits the data much better than the misspecified single-tree model for both the unlinked and linked models (bottom panel, Supplementary Fig. 1, Supplementary Fig. 3, and Supplementary Fig. 2D–F); the improvement in the fit of the true model increases (i.e., the difference in BIC becomes more negative) as the number of trees, the number of tips in each tree, and sequence length increases. This is expected because a single-tree model becomes an increasingly poor fit to data simulated under more trees.

**Figure 5:**
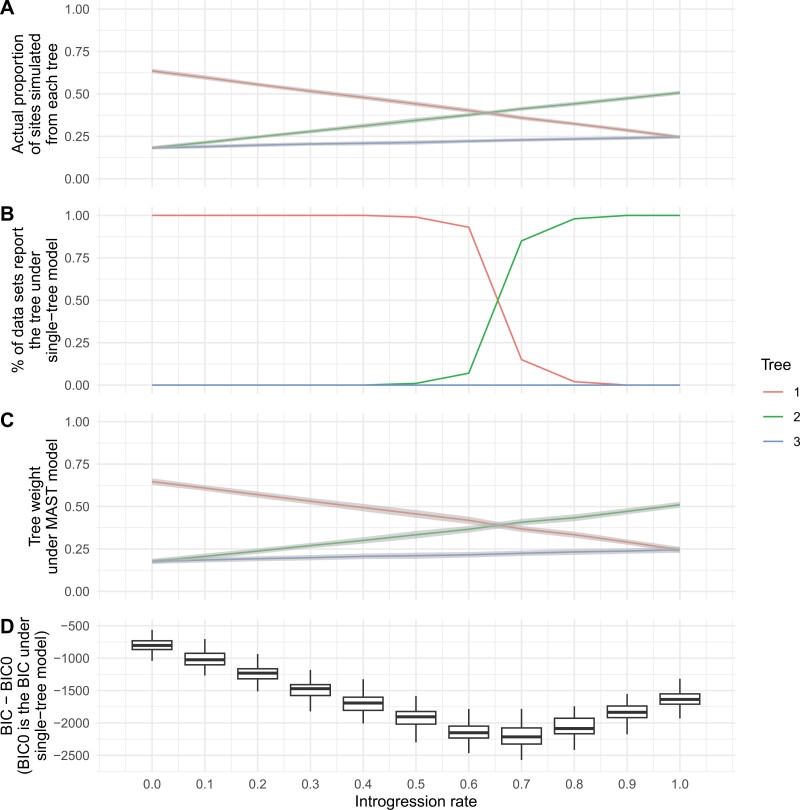
This figure compares the performance of the MAST model with the standard single-tree model using datasets simulated across introgression rates r ∈{0.0,0.1,...,1.0}. Specifically, it displays (a) the actual proportion of sites simulated under each tree for varying introgression rates. Mean values are represented by colored lines, while the gray regions indicate the standard deviation across the 100 datasets for each introgression rate; (b) Results from fitting the concatenated alignment to a single-tree model. At high introgression rates, the most probable tree topology shifts to *T*_E2_; (c) Tree weights estimated by the linked MAST model; (d) *BIC–BIC0*: the difference in BIC values between the linked MAST model (*BIC*) and the single-tree model (*BIC0*). A more negative difference between the BIC values of the MAST and single-tree models indicates a stronger preference for the MAST model over the standard single-tree model.

We also simulated scenarios with introgression, such that the minor trees are not expected to be equal in frequency. In these simulations, *T*_*E1*_ is the species tree (Supplementary Fig. 8) and increasing introgression makes topology *T*_*E2*_ increasingly frequent. When the introgression rate was between 0 and 0.6, *T*_*E1*_ is the optimal tree in the single-tree model (Fig. 5B) and the tree with the highest weight in the MAST model (Fig. 5C). When the introgression rate is above 0.6, in most datasets, the single-tree model and the MAST model reported *T*_*E2*_ as the optimal tree and the topology with the highest tree weight, respectively. Importantly, estimated weights from the MAST model closely match the proportion of sites simulated under each tree for different introgression rates (compare Fig. 5A to Fig. 5C). All these results are as expected from the simulations that were carried out (i.e., the topology matching the introgressed history does in fact become the most common). The MAST model is a much better fit when the tree topologies *T*_*E1*_ and *T*_*E2*_ are more equal in frequency, though it is a better fit across all of parameter space (because there is always ILS, even when there is no introgression, thus multiple trees are always a better fit to the data; Fig. 5D).

### Simulation 4–6: The MAST Model is Robust to the Inclusion of Trees with No Support in the Underlying Data

To test the robustness of the MAST model to the inclusion of incorrect additional topologies, we simulated data under a single topology but fit the data under a MAST submodel 1 (simulation 4) and MAST submodel 6 (simulation 5) with up to 10 topologies. The results show that with both MAST submodel 1 (Supplementary Fig. 4A–C) and MAST submodel 6 (Supplementary Fig. 5A), the true tree (which was always one of the trees included in the MAST model) had the highest weight among all of the trees included in the MAST model in the majority of simulations regardless of the simulation conditions when the sequences are long.

These simulations reveal some of the fundamental limitations of the MAST model to distinguish among very similar trees. When incorrect trees included in the MAST model were sufficiently different from the true tree (i.e., when the SPR distance of each incorrect tree in the MAST model was 2 or 3 SPR moves from the true tree), the percentage of simulations for which the true tree had the highest weight remained relatively high (i.e., over 80%) regardless of the other simulation conditions. However, when the incorrect trees included in the MAST model were close to the true tree (i.e., when they differed from the true tree by a single SPR move), in the worst case, the percentage of simulations for which the true tree had the highest weight dropped to, for submodel 1, 31% for 5K sequence length; 36% for 10K; and 51% for 50K, and, for submodel 6, 67% for 100K (Supplementary Fig. 4A–C; Supplementary Fig. 5A). This general trend is expected, because more similar trees will share more branches in common, making it more difficult for any model to distinguish between them. These results quantify some of the analytical limits of multitree mixture models as currently implemented. On the other hand, importantly, the inclusion of incorrect trees in the MAST model always led to large increases in the BIC score, such that researchers using this method to select the best model would reject the additional trees, and instead prefer the results from a single-tree model (Supplementary Fig. 4D–F; Supplementary Fig. 5B).

To evaluate the performance of the MAST submodel 6 when all the possible trees are included, we applied it with all 15 potential topologies to 100K-bp data sets simulated using two equally weighted 5-tip trees. On average, the MAST model reported that the weights of the true trees were 21.3% and 22.8%, while the weights of the other trees were at most 16.8 (Supplementary Fig. 6). More precisely, in 46%, 61%, and 73% of the simulations the two true trees were among the top 2, 3, and 4 trees with the highest tree weights. Sequentially adding trees to the MAST model shows that there is a big improvement (i.e., decrease) in the BIC value from the single-tree model to the MAST model with two true trees (Supplementary Fig. 7). After that, sequentially adding incorrect trees to the MAST model caused BIC values to worsen (i.e., increase; Supplementary Fig. 7). In 98% of the simulations, the MAST model with the two true trees was the optimal model according to the BIC value.

### Empirical dataset A: ILS in the Great Apes

**Figure 6:**
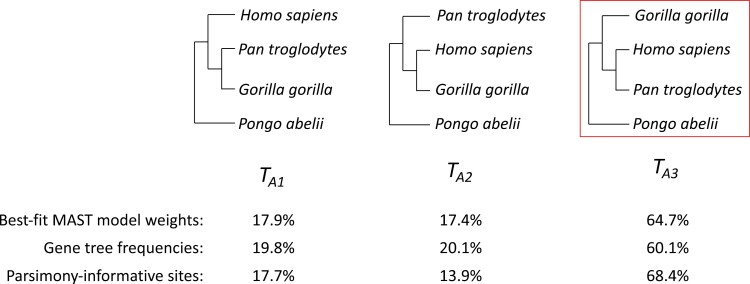
The 3 topologies for empirical dataset A. $T_{A3}$ is the commonly accepted species tree.

Figure 6 shows the 3 possible tree topologies $T_{A1}$, $T_{A2}$, and $T_{A3}$ for empirical dataset A, which is made up of 4 Great Apes (Table 1). We applied multiple methods to these alignments in order to estimate the frequency of the 3 tree topologies. Single-tree analyses applied to each gene separately reported 19.8%, 20.1%, and 60.1% of the genes with topologies $T_{A1}$, $T_{A2}$, and $T_{A3}$, respectively (Fig. 6; Supplementary Table 1). All MAST submodels reported similar tree weights of 17.9%, 17.4%, and 64.7% (Table 2). All methods find that the topology uniting human and chimpanzee has the highest weight, with the two minor topologies having approximately equal weights; these results are as expected from all previous analyses.

**Table 2:** Results of the empirical dataset A when applying IQ-Tree with a standard single-tree model and different MAST submodels with GTR + G substitution model

Model	Sub. matrix	Freqs.	RHAS	*T*_*A1*_	*T*_*A2*_	*T*_*A3*_	BIC
Single-tree						100.00%	4,978,549.51
MAST 1	unlinked	unlinked	unlinked	17.86%	17.40%	64.74%	4,975,971.28
MAST 2	unlinked	unlinked	linked	17.85%	17.44%	64.70%	**4,975,941.59**
MAST 3	unlinked	linked	unlinked	17.84%	17.48%	64.68%	4,978,121.95
MAST 4	unlinked	linked	linked	17.84%	17.48%	64.68%	4,978,097.70
MAST 5	linked	linked	unlinked	17.84%	17.48%	64.68%	4,977,961.91
MAST 6	linked	linked	linked	17.84%	17.48%	64.68%	4,977,938.91

Notes: There are 6 submodels of MAST representing different combinations of linked or unlinked substitution matrix (second column), nucleotide frequencies (third column), and rate heterogeneity across sites (fourth column). The fifth to seventh columns are the weights of the trees $T_{A1}$, $T_{A2}$, and $T_{A3}$. The eighth column lists the BIC values of different models. The bolded figure is the best BIC value which is from the MAST submodel 2.

The proportions of different topologies estimated by MAST are closer to the proportions of individual nucleotide sites from the genes supporting the various topologies than the percentage of gene trees (Supplementary Table 1). This may be because the weights of the MAST model more closely approximate the proportion of the sites in the alignment (instead of the percentage of loci) supporting different topologies. The BIC score from MAST submodel 2 was the best (Table 2), indicating that the MAST model with unlinked substitution model, unlinked frequencies, and linked RHAS was the best model among different MAST submodels for this dataset. Regardless, the BIC values of all MAST submodels were much lower than the BIC value reported by the single-tree model (Table 2), showing that a multitree mixture model had a much better fit to the data, and demonstrating the superiority of a multitree mixture model over a single-tree model when ILS causes gene tree discordance.

### Empirical Dataset B: Introgression in Macaques

**Figure 7:**
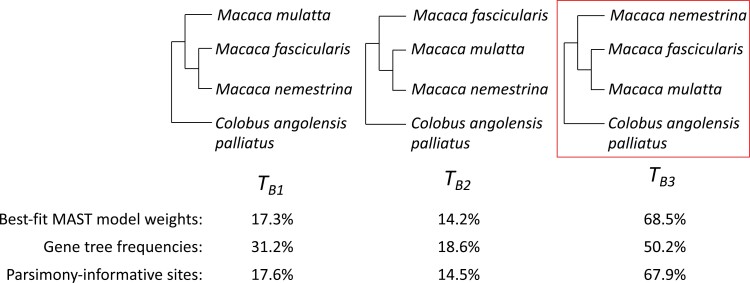
The 3 topologies for empirical dataset B. $T_{B3}$ is the commonly accepted species tree.

Figure 7 shows the 3 possible tree topologies $T_{B1}$, $T_{B2}$, and $T_{B3}$ for empirical dataset B, which is made up of multiple macaque species. Analyses of the individual gene trees using single-tree models for each locus revealed a large asymmetry in minor topologies (31.2%, 18.6%, and 50.2% for $T_{B1}$, $T_{B2}$, and $T_{B3}$_,_ respectively; Supplementary Table 2). However, both the proportions of parsimony-informative sites (17.6%, 14.5%, and 67.9% for $T_{B1}$, $T_{B2}$, and $T_{B3}$_,_ respectively; Supplementary Table 2) and the weights from the different MAST submodels (all around 17.3%, 14.2%, and 68.6% for $T_{B1}$, $T_{B2}$, and $T_{B3}$_,_ respectively; Fig. 7; Table 3) showed much more similar proportions and weights for the minor trees. Although the minor trees are still substantially different in frequency using the MAST analysis—consistent with introgression in this clade—the difference between them is much lower. Consistent with empirical dataset A, this result indicates that the gene tree frequencies are different from the frequencies reported by the MAST analysis, as the gene tree frequencies represent the proportions of genes supporting various topologies while the MAST tree weights are more closely related to the proportions of sites from the genes supporting different topologies.

**Table 3:** Results of the empirical dataset B when applying IQ-TREE with a standard single-tree model and different MAST submodels with GTR + G substitution model

Model	Sub. matrix	Freqs.	RHAS	*T*_*B1*_	*T*_*B2*_	*T*_*B3*_	BIC
single-tree						100.00%	4,906,941.36
MAST 1	unlinked	unlinked	unlinked	17.29%	14.15%	68.55%	4,905,832.06
MAST 2	unlinked	unlinked	linked	17.29%	14.19%	68.52%	**4,905,808.79**
MAST 3	unlinked	linked	unlinked	17.27%	14.24%	68.49%	4,906,632.17
MAST 4	unlinked	linked	linked	17.27%	14.25%	68.48%	4,906,605.01
MAST 5	linked	linked	unlinked	17.27%	14.24%	68.50%	4,906,651.67
MAST 6	linked	linked	linked	17.27%	14.23%	68.50%	4,906,633.71

Notes: There are 6 submodels of MAST, representing different combinations of linked or unlinked substitution matrix (second column), nucleotide frequencies (third column), and rate heterogeneity across sites (fourth column). The fifth to seventh columns are the weights of the trees $T_{B1}$, $T_{B2}$, and $T_{B3}$. The eighth column lists the BIC values of different models. The bolded figure is the best BIC value, which is MAST submodel 2.

### Empirical Dataset C: Great Apes + Macaques

Supplementary Figure 9 shows 9 tree topologies for empirical dataset C. This dataset combines the ingroup taxa from empirical datasets A and B, allowing us to test the accuracy of MAST when there are more possible topologies: the 9 topologies represent every combination of the 3 topologies present in each of empirical datasets A and B. The frequencies of the 9 tree topologies were similar across gene trees and sites in standard analysis (Supplementary Table 3) as well as largely similar to the results across MAST submodels (Table 4). MAST submodels 1 and 2 are the 2 best-fit models to the dataset according to the BIC values (Table 4), and both give tree weights that are relatively close to the corresponding tree weights for the respective analyses in empirical datasets A and B (Supplementary Tables 4 and 5). However, the results from the simpler submodel 2 (in which RHAS parameters are linked across classes) are closer to the expected values than those from submodel 1, which is likely due to the challenges of optimizing highly parameterized models.

**Table 4:** Results of the empirical dataset C when applying IQ-Tree with a standard single-tree model and different MAST submodels with GTR + G substitution model

Model	*T*_*C1*_	*T*_*C2*_	*T*_*C3*_	*T*_*C4*_	*T*_*C5*_	*T*_*C6*_	*T*_*C7*_	*T*_*C8*_	*T*_*C9*_	BIC
Single-tree									100.0%	5,187,194.8
MAST 1	0.4%	7.0%	8.4%	7.7%	2.9%	18.3%	13.0%	8.7%	33.6%	**5,183,982.5**
MAST 2	0.4%	10.4%	8.2%	2.1%	2.5%	14.0%	13.1%	8.4%	41.1%	5,183,988.4
MAST 3	0.2%	8.0%	5.2%	1.1%	0.2%	17.4%	15.2%	2.4%	50.4%	5,186,041.4
MAST 4	0.2%	0.2%	3.9%	0.6%	0.8%	29.3%	12.7%	19.8%	32.5%	5,185,924.7
MAST 5	0.0%	0.8%	9.8%	1.9%	0.4%	18.2%	17.1%	11.3%	40.4%	5,186,243.3
MAST 6	0.0%	0.7%	11.1%	1.9%	1.8%	20.7%	19.3%	8.4%	36.0%	5,186,194.1

Notes: Six submodels of MAST are for different combinations of linked or unlinked substitution matrix, nucleotide frequencies, and rate heterogeneity across sites. The second to tenth columns are the estimated tree weights between the topologies *T*_*C1*_, *T*_*C2*_, …, and *T*_*C9*_ for different MAST submodels. The bolded figure is the best BIC value among different submodels.

### Empirical Dataset D: Overcoming Known Biases in Concatenated ML

As mentioned, ML has a known bias toward symmetrical trees (Kubatko and Degnan 2007) when there is a large amount of underlying discordance and the true species tree is asymmetrical (i.e., *T*_*D1*_ or *T*_*D2*_ in Supplementary Fig. 10). Indeed, when analyzed under ML using a single-tree model, data from 4 Platyrrhine monkeys support a symmetrical tree (Table 5). In contrast, counts of genes trees and parsimony-informative sites support the asymmetrical tree *T*_*D1*_ as the species tree (Supplementary Table 6). Similarly, analyses using the MAST submodels also tended to return *T*_*D1*_ as the topology with the highest weight (Table 5). Among all the models, the MAST submodel 2 had the best BIC value, with reported tree weights 42.4%, 28.1%, and 29.6% for the topologies *T*_*D1*_*, T*_*D2*_*, T*_*D3*_, respectively. The tree weights are similar to the proportions of parsimony-informative sites from the genes that were inferred to support each of these topologies (i.e., 36.7%, 32.2%, 31.1%; Supplementary Table 6). It is notable that two MAST models estimated different trees with the highest weights (submodels 3 and 4; Table 5), though submodel 2 has a much lower BIC value than either of these. Overall, these results suggest that the MAST model is able to analyze a concatenated alignment using ML, but without the biases that come with the single-tree assumption.

**Table 5:** Results of the empirical data D when applying IQ-Tree with a standard single-tree model and different MAST submodels with GTR + G substitution model

Model	Sub. matrix	Freq.	RHAS	*T*_*D1*_	*T*_*D2*_	*T*_*D3*_	BIC
Single-tree	–	–	–			100.0%	6,185,094.0
MAST 1	unlinked	unlinked	unlinked	40.3%	23.0%	36.8%	6,177,609.0
MAST 2	unlinked	unlinked	linked	42.4%	28.1%	29.6%	**6,177,535.7**
MAST 3	unlinked	linked	unlinked	3.5%	4.7%	91.8%	6,182,942.1
MAST 4	unlinked	linked	linked	2.1%	81.3%	16.7%	6,182,954.3
MAST 5	linked	linked	unlinked	42.4%	32.0%	25.6%	6,184,689.7
MAST 6	linked	linked	linked	42.4%	32.0%	25.5%	6,184,618.7

Six submodels of MAST are for different combinations of linked or unlinked substitution matrix (second column), nucleotide frequencies (third column), and rate heterogeneity across sites (fourth column). The fifth, sixth, and seventh columns are the estimated tree weights between the topologies *T*_*D1*_, *T*_*D2*_, and *T*_*D3*_ for different MAST submodels, respectively. The bolded figure is the best BIC value among different submodels.

## Discussion

We have introduced the MAST model, which assumes that sites in a concatenated alignment may have evolved from a mixture of trees. This flexible assumption allows the method to be applied to the alignments that include multiple tree topologies, which is presumably true of almost any large dataset from a recombining genome. The implementation of the method allows different combinations of linked and unlinked parameters when estimating the substitution matrix, nucleotide, or AA frequencies, and the RHAS across different trees. This flexibility allows researchers to have many of the advantages of concatenated analyses—for example, a large amount of data and accurate estimate of complex substitution processes—while still incorporating gene tree heterogeneity, but without the need to make assumptions about the existence and location of putatively non-recombining loci. As such, the MAST model opens up the opportunity to study topological discordance in deep time, past the point where information from small, non-recombining gene tree alignments can be informative about relationships (Bryant and Hahn 2020).

Our simulations show that parameter estimates using the MAST model are reliable under a wide range of scenarios. In general, the ability of the MAST model to accurately estimate parameters depends on the balance between the amount of information in the data (e.g., the length, depth, and informativeness of the alignment), the number of parameters being estimated (e.g., the number of trees used in the model, and represented in the underlying alignment), and scale of the differences between the underlying tree topologies. Unsurprisingly, the MAST model performs best with long, informative alignments of many taxa, when the number of true trees is small, and when the differences between the underlying tree topologies is large. Nevertheless, our simulations show that the MAST model usually estimates tree weights with acceptably low error rates, even when the simulation conditions are more challenging, and the model is misspecified. Indeed, we show that by using standard approaches like the BIC, it is usually possible to identify the true trees that represent the data, even when these are not known in advance. Of course, these results do not prove the general identifiability of the model. The identifiability of parameters in complex models, like mixture models, has been addressed previously (Allman et al. 2011, Rhodes and Sullivant 2012). Rhodes and Sullivant (2012) gave an upper bound on the number of classes that ensures the generic identifiability of trees in models with a multi-tree mixture. Their method was based on the mixtures from different trees, provided that all the topologies share a certain type of common substructure in which a tripartition A|B|C exists such that the splits A | B∪C and A∪C | B are compatible with all trees. Parameters in the multi-tree mixture model are generically identifiable provided $m<\;k{\;}^{\;j-1}$ where *m* is the number of classes, *k* is the number of states (i.e., 4 for nucleotides; 20 for AAs), and the number of taxa in the partition A and in the partition B is both greater than or equal to *j*. However, establishing the identifiability of model parameters when there is no commonality between the trees remains an open problem (Rhodes and Sullivant 2012).

In order to use the MAST model to perform an analysis, the user must input a set of pre-specified tree topologies. A rooted three-taxon tree has only 3 possible topologies, but the number of topologies grows super-exponentially with the number of tips (Table 3.1 in Felsenstein 2003). This means that it will usually not be feasible to specify all possible topologies that exist in a moderate-sized dataset; for example, in empirical dataset D we only studied 3 of 15 possible topologies. This limits the model’s applicability. However, there are instances where researchers may want to focus on a narrower range of topologies of particular significance. For instance, even in a tree with 100 species, it may be the relationships among a smaller number of clades that are relevant: if ILS only occurs on one branch of the tree, then there are 3 relevant alternative topologies, no matter the number of total tips. In general, we recommend that users specify known alternative hypotheses—or carry out an exploratory analysis of individual gene trees—in order to choose a manageable set of topologies as input to the MAST model.

There are multiple known biases when carrying out concatenated analyses under the “treelikeness” assumption. As mentioned in the “Introduction” section, single-tree concatenated ML is statistically inconsistent in the presence of large amounts of discordance: it will return the incorrect tree with increasing probability as more data are added (Kubatko and Degnan 2007). Our analyses of Platyrrhine monkeys suggest that the MAST model can solve this problem, giving the highest weight to the topology favored by other (statistically consistent) methods. In addition to inferring the wrong tree topology, the branch lengths inferred from concatenated analyses are biased in the presence of discordance (Mendes and Hahn 2016; Ogilvie et al. 2017). Such biases can lead to misestimation of divergence times when using the entire concatenated alignment. The MAST model allows researchers to estimate the branch lengths of individual topologies—we, therefore, recommend estimating divergence times using branch lengths obtained from the topology matching the species tree. While these times still represent genic divergence (and not species divergence; Edwards and Beerli 2000), they will be free of the bias associated with single-tree concatenation.

The output of our method is a set of weights associated with each input tree topology. Although the MAST model is not based on a particular biological model of discordance (e.g., the MSC or MSNC), we expect that the estimated weights should correspond to biologically relevant features of the data. Both our analyses of simulated and empirical data revealed that MAST gives the highest weight among all input trees to the tree that occurs most frequently in the gene trees. This is expected since the MAST weight will be most heavily influenced by the proportion of sites that are associated with each input tree. We note, however, that the highest-weight tree from MAST may not be the species tree (just as the most frequent gene tree may not correspond to the species tree (Degnan and Rosenberg 2006)). Moreover, the reported weights in the MAST model are highly correlated with the proportion of phylogenetically informative sites that support each tree. This correlation is expected because the likelihood of each site is calculated as the weighted sum of the likelihood of the site over all the trees (Equation (1)) and the overall likelihood value is the product of the likelihoods over all the sites. This result, together with the accurate estimation of minor tree weights, means that we can use these estimates to infer introgression from MAST output. Common tests for introgression are based on the expectation that the two minor trees are equal in frequency (e.g., the “ABBA-BABA” test; Green et al. 2010). One post hoc approach to inferences of introgression using MAST would be to test for a significant difference in the weights supporting each of two minority trees. Alternatively, it should be possible to compare the likelihoods of models that either link or unlink the weights of the minority trees. Greater support for the unlinked model would indicate that the two trees are not equal in frequency, and would support an inference of introgression. Such an approach would be of great benefit to testing for introgression deeper in time, where individual phylogenetically informative sites and individual gene trees may not be accurate enough to make strongly supported inferences about introgression (Vanderpool et al. 2020).

The MAST model is a flexible phylogenetic approach that models situations in which the sites of an alignment have evolved under multiple bifurcating tree topologies. Each tree has its own topology, a separate set of branch lengths, a substitution model, a set of nucleotide or amino-acid frequencies, and a rate heterogeneity model. However, there are still some limitations to the current implementation. In addition to the future directions mentioned above, we would like to extend the MAST model to (i) perform a tree topology search for an input number of trees, thus relaxing the requirement that the user must pre-specify topologies; (ii) be able to compute the optimal number of trees needed to represent the input dataset, relaxing the requirement that the user specifies the number of trees ahead of time; and (iii) find the best set of substitution models and RHAS models for each tree separately. These directions are challenging but will be useful in analyzing genome-scale datasets at any evolutionary timescale.

## supplementary material

Data available from the Dryad Digital Repository: https://doi.org/10.5061/dryad.51c59zwfx

## Data Availability

Data, scripts, and supplementary materials are available from the Dryad Digital Repository: https://doi.org/10.5061/dryad.51c59zwfx MAST model has been implemented in IQ-Tree2, which is available in the Github: https://github.com/iqtree/iqtree2/releases/tag/v2.2.0.7.mx
